# GO2Vec: transforming GO terms and proteins to vector representations via graph embeddings

**DOI:** 10.1186/s12864-019-6272-2

**Published:** 2020-02-18

**Authors:** Xiaoshi Zhong, Rama Kaalia, Jagath C. Rajapakse

**Affiliations:** School of Computer Science and Engineering, Nanyang Technological University, Singapore, Singapore

**Keywords:** Graph embeddings, Vector representations, Gene ontology, CESSM evaluation, Protein-protein interaction prediction

## Abstract

**Background:**

Semantic similarity between Gene Ontology (GO) terms is a fundamental measure for many bioinformatics applications, such as determining functional similarity between genes or proteins. Most previous research exploited information content to estimate the semantic similarity between GO terms; recently some research exploited word embeddings to learn vector representations for GO terms from a large-scale corpus. In this paper, we proposed a novel method, named GO2Vec, that exploits graph embeddings to learn vector representations for GO terms from GO graph. GO2Vec combines the information from both GO graph and GO annotations, and its learned vectors can be applied to a variety of bioinformatics applications, such as calculating functional similarity between proteins and predicting protein-protein interactions.

**Results:**

We conducted two kinds of experiments to evaluate the quality of GO2Vec: (1) functional similarity between proteins on the Collaborative Evaluation of GO-based Semantic Similarity Measures (CESSM) dataset and (2) prediction of protein-protein interactions on the Yeast and Human datasets from the STRING database. Experimental results demonstrate the effectiveness of GO2Vec over the information content-based measures and the word embedding-based measures.

**Conclusion:**

Our experimental results demonstrate the effectiveness of using graph embeddings to learn vector representations from undirected GO and GOA graphs. Our results also demonstrate that GO annotations provide useful information for computing the similarity between GO terms and between proteins.

## Background

**Fig. 1:**
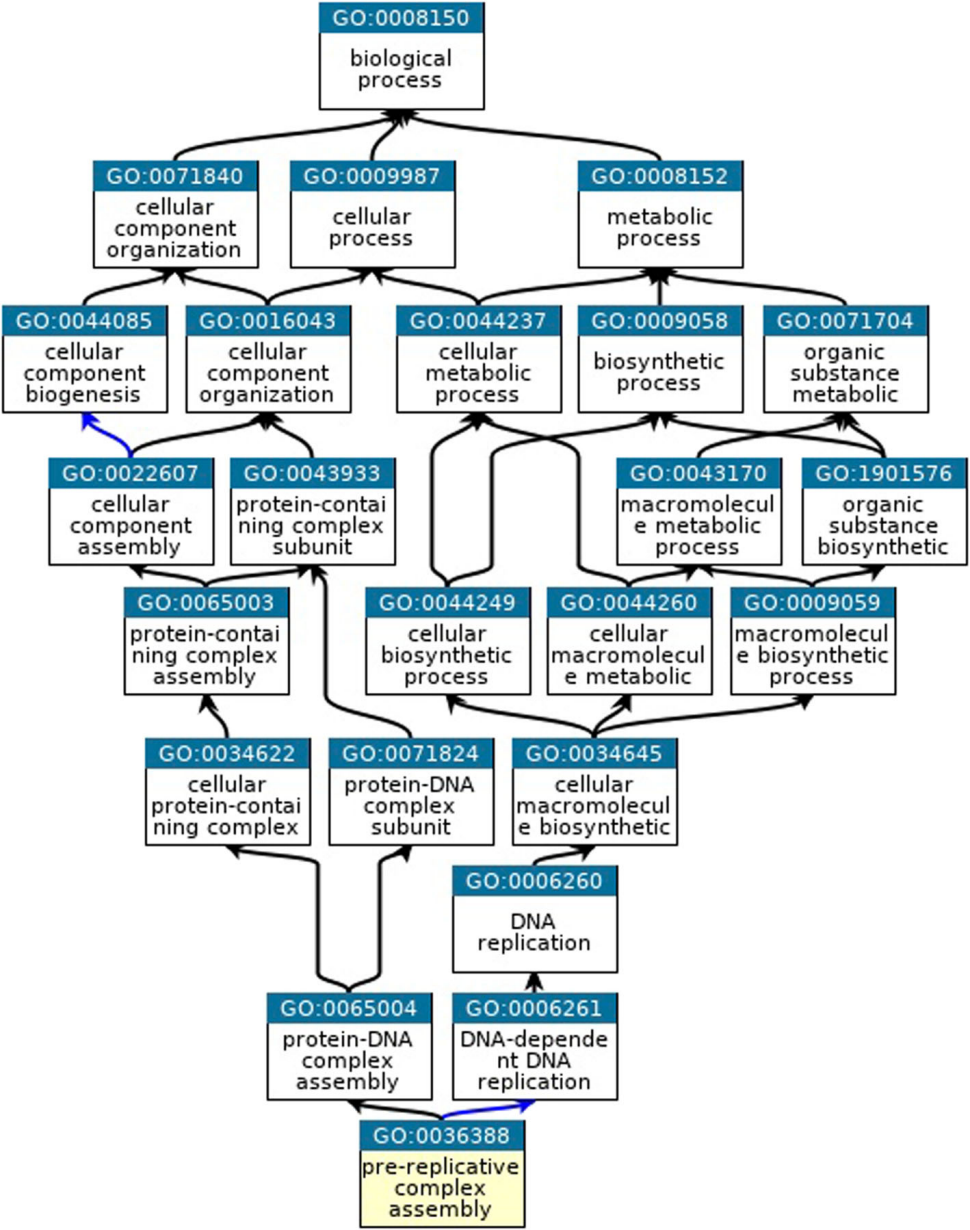
GO graph for term “GO:0036388 (pre-replicative complex assembly)” (adapted from https://www.ebi.ac. uk/QuickGO/term/GO:0036388)

Gene Ontology (GO) provides a set of structured and controlled vocabularies that describe gene products and molecular properties [1]. GO includes three categories of ontologies: Biological Process (BP), Cellular Component (CC), and Molecular Function (MF); each category of the ontologies is organized as a directed acyclic graph (DAG) and is referred to as a GO graph, where a node denotes a GO term while an edge denotes a kind of relationships between two GO terms. GO terms are defined in a hierarchy with a root node at the top, and child GO terms are related to parent GO terms via three main kinds of relationships: “ *is*_*a*”, “ *part*_*of*”, and “*regulates*.” GO describes complex biological phenomenon and accordingly intones a complex hierarchy. A parent node may have more than one child and a child node may have more than one parent and different relations to its different parents. Figure 1 shows a part of GO graph of the BP category for the term “GO:0036388 (pre-replicative complex assembly)”, where “GO:0036388” is its term ID and “pre-replicative complex assembly” is its descriptive axiom. The term “GO:0036388” can be traced to the root term “GO:0008150 (biological process).”

GO terms have been used to annotate many biomedical databases (e.g., model organism database (MOD) [2], UniProt [3], and SwissProt [4]) and interpret meanings of biomedical entities and experiments, such as genetic interactions, functional interactions, protein-protein interactions, biological pathways, and disease similarities. The importance of GO terms leads their semantic similarity to be crucial for many bioinformatics research and applications. Therefore, computing the semantic similarity of GO terms has attracted tremendous attention.

Most previous methods of estimating the semantic similarity of GO terms are based on the information content (IC). Such pioneered methods [5–7] and their variants [8–24] compute the semantic similarity between two GO terms according to their distances to the closest common ancestor term associated with the structure of GO DAG or associated statistics of their common ancestor terms. These methods have succeeded in the development of computing the GO term similarity over the past two decades.

Recently, some researchers employed word embeddings, which have been developed in the area of natural language processing, to learn vectors representing GO terms and proteins, and use the learned vectors to compute the semantic similarity between GO terms and the functional similarity between proteins [25–27]. These methods mainly use the word2vec model [28] to learn vectors for each word from the corpus derived from the descriptive axioms of GO terms and proteins (e.g., “pre-replicative complex assembly”); thereafter, the vectors of words are combined into vectors of GO terms and proteins according to the words in the descriptive axioms of GO terms and proteins.

In this paper, we proposed a method to compute the semantic similarity of GO terms by transforming a GO graph into vector representations by using a graph embeddings technique. Specifically, we first transformed a GO graph into vector representations where each node in the GO graph is represented by a vector of low-rank features. After that transformation, the semantic similarity of GO terms and the functional similarity of proteins are computed by the cosine distance of their corresponding vectors. Graph embeddings are capable of capturing the structural information connecting the nodes in the entire GO graph. On the one hand, when compared with the structure-based information content methods, which mainly consider the nearest common ancestors of two nodes, graph embeddings consider the information from every path between two nodes. Graph embeddings therefore can fully portray the relationship of two nodes in the entire graph. On the other hand, when compared with the corpus-based methods, including the traditional information content methods and the word embedding methods, graph embeddings can employ the expert knowledge stored in the graphical structure. In our experiments, we use the node2vec model [29] as the representative of graph embedding techniques. The node2vec model adopts a strategy of random walk over an undirected graph to sample neighborhood nodes for a given node, and preserves both neighborhood properties and structural features. As far as we know, the node2vec model has not been applied to computer the protein similarity by using GO graph.

There are several ways to evaluate the quality of the semantic similarity between GO terms and of the functional similarity between proteins. One way is to compare them with human similarity ratings [7, 15, 30] to see the correlation between computational results and human annotation results. An alternative way is to evaluate the quality of downstream applications using the semantic similarity between GO terms and the functional similarity between proteins. In this paper, we used two kinds of downstream tasks to evaluate GO2Vec. One task is the similarity of proteins on the Collaborative Evaluation of GO-based Semantic Similarity Measures (CESSM) [31], which provides an interface for researchers to evaluate their similarity measures of proteins with different ones on a standard dataset (comprising 13,430 pairs of proteins from 55 types of organisms). The other is the prediction of protein-protein interactions (PPI) on two kinds of datasets, Yeast and Human PPI networks [32]. Experimental results demonstrate the effectiveness of GO2Vec over the information content-based methods (i.e., Resnik [7], Lin [6], Jang&Conrath [5], simGIC [33], and simUI [34]) and the word embedding-based methods (i.e., Onto2Vec [25] and w2vGO [27]).

## Results

We conducted two kinds of experiments to evaluate the quality of the learned vectors of GO2Vec: (1) evaluation of protein similarities on the CESSM dataset and (2) prediction of protein-protein interactions (PPI) on Yeast and Human networks. The results of GO2Vec are compared with information content-based methods (i.e., Resnik [7], Lin [6], Jang&Conrath [5], simGIC [33], and simUI [34]) and corpus-based vector representation methods (i.e., Onto2Vec [25] and w2vGO [27]). The technical details of GO2Vec and the compared methods are described in next section.

### Gene ontology and GO annotations

The Gene Ontology [1] includes three independent categories of ontologies: BP, CC, and MF. The BP ontology includes GO terms that describe a series of events in biological processes. The CC ontology includes GO terms that describe molecular events in the components of a cell. The MF ontology includes GO terms that describe the chemical reactions (e.g., catalytic activity and receptor binding). These GO terms have been used to annotate biomedical entities (e.g., genes and proteins) and interpret biomedical experiments (e.g., genetic interactions and biological pathways). Table 1 summarize the statistics of the three GO graphs.

**Table 1:** Statistics of GO graphs. ‘#Terms’ denotes the number of GO terms while ‘#Edges’ denotes the number of edges

Ontology	#Terms	#Edges
BP	30,705	71,530
CC	4,380	7,523
MF	12,127	13,658

In each kind of experiments, we obtained the GO annotations by mapping the proteins to the UniProt database [3]. Generally, a protein is annotated by several GO terms. For example, the protein ‘P06182’ is annotated by the GO terms ‘GO:0004408’, ‘GO:0005743’, ‘GO:0005758’, ‘GO:0018063’, ‘GO:0046872’.

### Protein similarity on CESSM

In this kind of experiment, we aimed to evaluate the quality of the learned vectors by computing the functional similarity between proteins on the CESSM dataset [31]. We compare the results with the representative information content based methods, namely Resnik [7], Lin [6], Jang&Conrath [5], simGIC [33], and simUI [34], and the corpus-based vector presentation method w2vGO [27].

#### CESSM dataset

CESSM [31] provides an interface with 13,430 pairs of proteins for researchers to compare their functional similarity measures of proteins. The 13,430 pairs of proteins include 1,039 unique proteins, which are collected from 55 types of organisms (e.g., HUMAN and YEAST). We get the organism information by mapping the proteins to the UniProt database. These proteins are diverse enough to evaluate the robustness of the measures for the semantic similarity between proteins. CESSM provides three kinds of combinations for the Resnik, Lin, and Jang&Conrath methods: average [8], maximum [11], and best-match average [14]. The best-match average method achieves the best performance in all the three methods. In this paper, we report their performance under the best-match average method.

#### Experiments

**Fig. 2:**
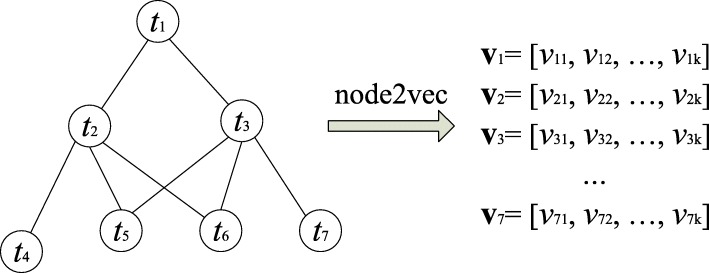
GO2Vec on GO graph: using GO terms and term-term relations. node2vec is applied on the GO graph to transform the notes to vectors. *t*_*i*_ denotes a GO term and **v**_*i*_ denotes its *k*-dimensional vector, where *v*_*ij*_ is the *j*-th element of **v**_*i*_

**Fig. 3:**
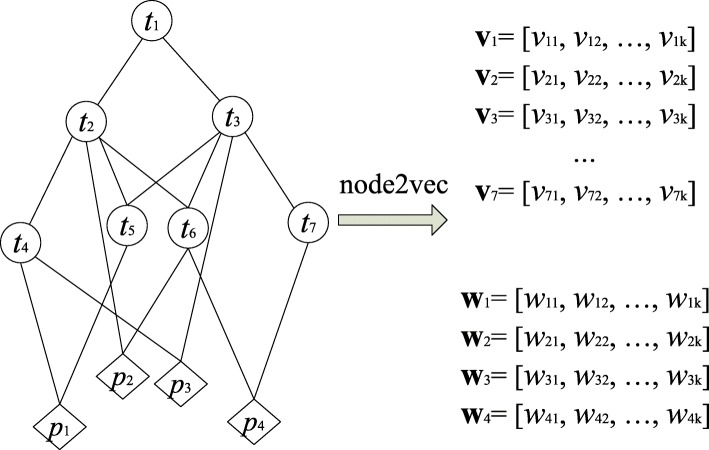
GO2Vec on GOA graph: using GO terms, term-term relations, and term-protein annotations. The denotations of *t*_*i*_ and **v**_*i*_ are the same as the ones in Fig. 2. *p*_*m*_ denotes a protein and **w**_*m*_ denotes its *k*-dimensional vector, where *w*_*mn*_ is the *n*-th element

We followed CESSM’s setting to use each category of GO ontologies (i.e., BP, CC, and MF) for the GO and GOA graph transformations as well as the semantic similarity computation of GO terms and proteins. For the GO graph transformation of each GO ontology, we do not use the GO annotations (see Fig. 2). For the GOA graph transformation, we used the GO graph with the GO annotations to form a graph (see Fig. 3).

For the node2vec model, we applied its code in our experiments by trying different settings for the parameters and report the best performance. The setting that achieves the best results is as follows: 100 dimensions, 20 walks per node, 100-length per walk and 20 walks per node, undirected binary edges.

We implemented several versions of GO2Vec to computer the functional similarity between proteins on both the GO and GOA graphs in both ways described in “Functional similarity between proteins” section. The version that uses the modified Hausdorff distance (see Eq. (9)) on the GO graph transformation is denoted by ‘GO2Vec_mhd_go’; the version that uses the cosine distance (see Eq. (7)) on the GOA graph transformation is denoted by ‘GO2Vec_cos_goa’; the version that uses the modified Hausdorff distance on the GOA graph transformation is denoted by ‘GO2Vec_mhd_goa’.

The performance of protein similarity on CESSM is evaluated under two metrics: *ECC* similarity [35] and *Pfam* similarity [36]. The *ECC* similarity is computed by the Enzyme Comparison Class (ECC) metric [35]. The *Pfam* similarity is computed through the Jaccard measure, indicating the similarity between two proteins by the ratio between the number of *Pfam* families [37] they share and the total number of *Pfam* families they have.

Table 2 reports the overall performance of our models and the compared models on the CESSM dataset; the best result in each similarity metric is highlighted in boldface. Except on the MF ontology under the *Pfam* metric, GO2Vec achieves the best performance on all the three ontologies in the two metrics. Specifically, GO2Vec_mhd_goa achieves four best results and GO2Vec_mhd_go achieves one best result. This indicates that graph embeddings can better capture information that is useful for computing the semantic similarity of GO terms and proteins compared to information content-based methods and corpus-based word embeddings method.

**Table 2:** Performance of our models and the compared models on the CESSM dataset

Metric	Model	BP	CC	MF
ECC	Resnik	0.4258	0.3444	0.4842
	Lin	0.4217	0.3391	0.5162
	Jang&Conrath	0.4114	0.2520	0.5189
	simGIC	0.3888	0.3503	0.5875
	simUI	0.3818	0.3527	0.5783
	w2vGO	0.4204	0.3516	0.4905
	GO2Vec_mhd_go	0.4476	**0.3650**	0.6715
	GO2Vec_cos_goa	0.4251	0.3507	0.6472
	GO2Vec_mhd_goa	**0.4508**	0.3618	**0.6792**
Pfam	Resnik	0.4507	0.4676	0.5221
	Lin	0.3811	0.4562	0.5149
	Jang&Conrath	0.2741	0.3321	0.4503
	simGIC	0.4383	0.4682	**0.5825**
	simUI	0.4253	0.4873	0.5504
	w2vGO	0.4569	0.4735	0.5436
	GO2Vec_mhd_go	0.5041	0.4902	0.4537
	GO2Vec_cos_goa	0.4916	0.4727	0.4315
	GO2Vec_mhd_goa	**0.5118**	**0.4975**	0.4453

The best result in each metric is highlighted in boldface

Let us look at the comparison between using the GOA graph and using the GO graph. The models that use a GOA graph achieves better performance than the ones that use a GO graph in most ontologies in most evaluation metrics. This indicates that GO annotations provide useful information for computing the semantic similarity between GO terms and the functional similarity between proteins.

### PPI prediction on yeast and human datasets

In this experiment, we aimed to evaluate the learned vectors by predicting the protein-protein interactions in two species whose datasets are collected from the STRING database [32]: Yeast and Human. We compare our method with the representative information content-based methods, namely Resnik [7], Lin [6], Jang&Conrath [5], and simGIC [33], and the corpus-based word embeddings method Onto2Vec [25].

#### PPI datasets

We got from the STRING database [32] two datasets for protein-protein interactions (v11.0 version): Yeast (Saccharomyces cerevisiae) and Human (Homo sapiens). The Yeast dataset contains 3287 proteins and 1,845,966 interactions while the Human dataset contains 9677 proteins and 11,759,455 interactions. We mapped the proteins to the UniProt database and filter out those proteins that could not be found in the UniProt database and discard those interactions involving filtered proteins. After filtering, there remains 2851 yeast proteins and 6966 human proteins. We sampled 25,000 yeast interactions and 1,000,000 human interactions from the remaining interactions as positive instances for experiments. From the remaining proteins, we sampled the same number of pairs of proteins, between which there is no interaction, as negative instances for experiments. In total, we had 50,000 pairs of yeast proteins and 2,000,000 pairs of human proteins for PPI prediction experiments.

We followed the setting of Onto2Vec to merge all the three categories of ontologies and GO annotations into a large graph. Like the CESSM experiment, we also implement several versions of GO2Vec for the PPI prediction, and their denotations are the same as those used in CESSM experiments. See “Experiments” section for details on the setup.

For the node2vec model, we used the same setting as the one used in the first kind of experiment.

The performance of PPI prediction is evaluated under the metric of area under the ROC curve (AUC), where ROC stands for the receiver operating characteristic, which is widely used to evaluate the performance of classification and prediction tasks. ROC is defined by the relation between the true-positive rate (TPR) and the false-positive rate (FPR). TPR is defined as $TPR=\frac {TP}{TP+FN}$ and FPR is defined as $FPR=\frac {FP}{FP+TN}$, where *TP* denotes the number of true positives, *FP* the number of false positives, *TN* the number of true negatives, and *FN* the number of false negatives.

Table 3 reports the overall performance of our models and the compared models on the two PPI datasets; the best result in each dataset is highlighted in boldface. (The results of the compared models are reported directly from the paper of Onto2Vec [25]. Onto2Vec implemented several variants and we here reported their average performance.) GO2Vec achieves the best performance on the two PPI datasets. This indicates again that graph embeddings can capture structural information from graph that is useful for computing the semantic similarity between GO terms and the functional similarity between proteins.

**Table 3:** AUC of the ROC curve for PPI prediction on Yeast and Human datasets

Model	Yeast	Human
Resnik	0.7942	0.7891
Lin	0.7354	0.7222
Jang&Conrath	0.7108	0.7027
simGIC	0.7634	0.7594
Onto2Vec	0.7660	0.7593
GO2Vec_mhd_go	0.8026	0.7953
GO2Vec_cos_goa	0.7824	0.7676
GO2Vec_mhd_goa	**0.8154**	**0.8046**

The best result in each metric is highlighted in boldface

Compare the performance of using the GO graph and using the GOA graph. GO2Vec_mhd_goa performs better than GO2Vec_mhd_go in both datasets. This indicates again that GO annotations successfully provide useful information for computing the semantic similarity between GO terms and the functional similarity between proteins.

## Discussion

There are two potential limitations in our method. First, GO2Vec transforms directed graphs into undirected graphs, which might result in a loss of structural information. Second, GO2Vec treats the edges of term-term relations and term-protein annotations equal in a GOA graph, while the term-term relations and the term-protein annotations might not be equal in reality. We will investigate the two issues in our future work.

## Conclusion

In this paper, we employed the technique of graph embeddings to transform the GO and GOA graphs into vector representations so as to compute the semantic similarity between GO terms and the functional similarity between proteins in an Euclidean space. To evaluate the quality of our method, we conducted two kinds of experiments, namely protein similarity on the CESSM dataset and protein-protein interaction prediction, and compared our method with the traditional information content-based methods and the recent corpus-based word embedding methods. Experimental results demonstrate the effectiveness of using graph embeddings to learn vector representations from GO and GOA graphs. Experiments also demonstrate that GO annotations provide useful information for computing the similarity between GO terms and between proteins.

## Methods

Recent years have witnessed an advancement of unsupervised feature learning from sequences of words (e.g., word2vec [28, 38] and GloVe [39]) and graphs (e.g., DeepWalk [40], LINE [41], and node2vec [29]) in the fields of data mining and natural language processing. These works propose to learn latent vector representations of words in a corpus or nodes in a graph, and have achieved considerable success in many tasks, such as language modelling, text classification, syntactic parsing, and social network analysis. In this paper, we used the techniques of graph embeddings to transform the nodes in a GO graph and a GO annotation graph into vector representations in order to evaluate the similarity between GO terms and proteins. There are three pioneered models of graph embeddings, namely DeepWalk [40], LINE [41], and node2vec [29]. Since node2vec achieves better performance in our experiments, we used the node2vec model in this paper.

### node2vec

Let (*T*,*E*) represent a graph where *T* denotes the set of nodes and *E*⊆(*T*×*T*) denotes the set of edges. The goal is to learn a mapping function $f: T \rightarrow \mathbb {R}^{k}$ that transforms the nodes to vector representations in the space *ℝ*^*k*^, where the parameter *k* specifies the dimensions of the vector representations. *f* can be represented by a matrix of parameters with the size |*T*|×*k*. For each node *t*∈*T*, *N*(*t*)⊂*T* denotes the set of neighbourhood nodes of node *t*, generated through a sampling strategy.

The node2vec model aims to optimize Eq. (1), which maximizes the log-probability of observing a network neighborhood *N*(*t*) for a node *t* conditioned on its vector representation, given by *f*.

1 $$  \max_{f} \sum_{t \in T} \log P(N(t)|f(t))  $$

To make the optimization problem resolvable, the node2vec model makes two assumptions:

Conditional independence: given the vector representation of the source node *t*, the likelihood of observing a neighborhood node *t*^′^ is independent of observing any other neighborhood node. This is expressed by Eq. (2).

2 $$  P(N(t)|f(t)) = \prod_{{t}' \in N(t)} P({t}'|f(t))  $$

Symmetry in feature space: the source node *t* and the neighborhood node *t*^′^ have a symmetric effect on each other in the feature space. This makes the conditional likelihood of each pair of source-neighborhood nodes as a dot product of their features, defined by Eq. (3).

3 $$  P({t}'|f(t)) = \frac{\exp(f({t}') \cdot f(t))}{\sum_{t^{\prime\prime} \in T} \exp(f(t^{\prime\prime}) \cdot f(t))}  $$

With the above two assumptions, (1) is simplified to (4):

4 $$  \max_{f} \sum_{t \in T} \left(\sum_{{t}' \in N(t)} f({t}') \cdot f(t) - \sum_{t^{\prime\prime} \in T} \exp(f(t^{\prime\prime}) \cdot f(t))\right)  $$

The problem of sampling neighborhoods of a source node is viewed as a problem of performing a local search. To achieve this, the node2vec model adopts a flexible sampling strategy that allows the model to smoothly interpolate between two extreme sampling strategies for generating neighborhood sets *N*(*t*): breadth-first sampling and depth-first sampling.

Given a source node *t*, the node2vec model simulates a random walk of fixed length *l*. Let *c*_*i*_ denote the *i*-th node in the walk, starting with *c*_0_=*t*. Node *c*_*i*_ is generated by the following distribution:

5 $$ P(c_{i}=x|c_{i-1}=t)=\left\{\begin{array}{ll} \frac{\pi_{tx}}{Z} & \text{if} (t,x) \in E \\ 0 & \text{otherwise} \end{array}\right.  $$

where *π*_*tx*_ is the transition probability between nodes *t* and *x*, and *Z* is the normalizing constant.

### GO graph and GOA graph to vector representations

Figures 2 and 3 illustrate the GO2Vec transformation of an undirected GO or GOA graph into vector representations. A GO graph includes only the term-term relations of GO terms, while a GOA graph includes both the term-term relations of GO terms and the term-protein annotations between GO terms and proteins. Since a protein is annotated by several GO terms, merging term-term relations and term-protein annotations into a graph enables the graph embedding models to capture the structural information from both term-term relations and term-protein annotations. During the transformation, GO2Vec first transforms a directed graph into an undirected graph by simply setting directed edges as undirected edges, and then applies node2vec on the undirected graph to transform the nodes into their vector representations. Transforming a directed graph to an undirected graph might result in a loss of some information. However, since node2vec adopts a strategy of random walks to sample neighborhood nodes for a given source node, and such strategy approximates diffusion on the graph and performs better on undirected graphs than directed graphs, we use undirected graphs in our experiments. We also observe that using undirected graphs achieves better performance than using directed graphs.

### Semantic similarity between GO terms

After using node2vec for transformation, each GO term is represented by a *k*-dimensional vector. We can then compute the semantic similarity of GO terms by computing the distance of their corresponding vectors. That is, **v**_*i*_ and **v**_*j*_ denote the vector representations of terms *t*_*i*_ and *t*_*j*_, respectively, the semantic similarity *sim*(*t*_*i*_,*t*_*j*_) between terms *t*_*i*_ and *t*_*j*_ is given by the distance *dist*(**v**_*i*_,**v**_*j*_) between their vectors **v**_*i*_ and **v**_*j*_ in the Euclidean space. The distance *dist* can be computed by the cosine distance:

6 $$ sim(t_{i}, t_{j})=cos(\mathbf{v}_{i}, \mathbf{v}_{j})=\frac{\mathbf{v}_{i} \cdotp \mathbf{v}_{j}}{||\mathbf{v}_{i}||||\mathbf{v}_{j}||}  $$

### Functional similarity between proteins

There are two ways to compute the semantic similarity of proteins. One way is directly through the learned vectors of proteins, similar to the one for the semantic similarity between GO terms. The other way is through the learned vectors of GO terms.

#### From learned vectors of proteins

Let **w**_*m*_ and **w**_*n*_ denote the learned vectors of protein *p*_*m*_ and *p*_*n*_. The functional similarity *fun*(*p*_*m*_,*p*_*n*_) between two proteins is defined by the cosine distance *cos*(**w**_*m*_,**w**_*n*_) of their corresponding vectors **w**_*m*_ and **w**_*n*_, as shown by Eq. (7).

7 $$  fun(p_{m}, p_{n})=cos(\mathbf{w}_{m}, \mathbf{w}_{n})=\frac{\mathbf{w}_{m} \cdotp \mathbf{w}_{n}}{||\mathbf{w}_{m}||||\mathbf{w}_{n}||}  $$

#### From learned vectors of GO terms

Since a protein is annotated by several GO terms under each category of GO graphs, we can view protein *p* as a set of GO terms that annotate *p*. Let *T*_*m*_ denote the set of GO terms that annotate protein *p*_*m*_, and *T*_*n*_ denote the set of GO terms that annotate protein *p*_*n*_. To compute the functional similarity between proteins *p*_*m*_ and *p*_*n*_, we need only to compute the semantic similarity of their sets of GO terms (i.e., *T*_*m*_ and *T*_*n*_). Since a set of GO terms can be represented by its corresponding set of vectors, the semantic similarity of two proteins can be computed by the distance of the two sets of vectors. Let **V**_*m*_ denote the set of vectors that correspond to *T*_*m*_, and **V**_*n*_ correspond to *T*_*n*_. Then, the functional similarity between two proteins is given by the semantic similarity between two sets of vectors, that is, the distance between the corresponding sets of vectors:

8 $$  fun(p_{m}, p_{n})=fun(T_{m}, T_{n})=dist(\mathbf{V}_{m}, \mathbf{V}_{n})  $$

There are several measures that can be used to compute the semantic similarity between two sets of vectors [17, 42]. In our experiments, we find that the modified Hausdorff distance [43] achieves much better performance than the linear combination of vectors. Therefore, in this paper we adopt the modified Hausdorff distance to compute the distance of two sets of vectors for the functional similarity betwwen two proteins.

Given two points in a vector space (e.g., the Euclidean space), *dist* measures the distance of the two vectors in the space. The smaller the *dist* score is, the closer the two vectors are. Since GO terms are transformed into vectors, the *dist*(**v**_*i*_,**v**_*j*_) score can be used to estimate the spatial relation of two GO terms *t*_*i*_ and *t*_*j*_. *dist*(**v**_*i*_,**v**_*j*_) is defined by the opposite of the distance function: the larger the *dist*(**v**_*i*_,**v**_*j*_) is, the closer the terms *t*_*i*_ and *t*_*j*_ are. Therefore, we get a variant of the modified Hausdorff distance [43] for computing the functional similarity between proteins *p*_*m*_ and *p*_*n*_ from two sets of vector representations of GO terms. Specifically, the modified Hausdorff distance of two proteins is defined by *fun*(**V**_*m*_,**V**_*n*_)=

9 $$ \begin{aligned} \min&\left\{\frac{1}{|\mathbf{V}_{m}|}{\sum}_{\mathbf{v}_{m}\in\mathbf{V}_{m}}\max_{\mathbf{v}_{n}\in\mathbf{V}_{n}}dist(\mathbf{v}_{m}, \mathbf{v}_{n}),\right.\\ &\left.\frac{1}{|\mathbf{V}_{n}|}{\sum}_{\mathbf{v}_{n}\in\mathbf{V}_{n}}\max_{\mathbf{v}_{m}\in\mathbf{V}_{m}}dist(\mathbf{v}_{m}, \mathbf{v}_{n}) \right\} \end{aligned}   $$

where |**V**_*m*_| denotes the number of vectors in **V**_*m*_. In Eq. (9), *dist*(**v**_*m*_,**v**_*n*_) denotes the distance of two vectors, and in this paper, we use the cosine distance: *dist*(**v**_*m*_,**v**_*n*_)=*cos*(**v**_*m*_,**v**_*n*_).

The first way can be only used in the GOA graph transformation while the second way can be used in both the GO and GOA graph transformations.

### Information content-based methods

Resnik’s semantic similarity is based on the information content (IC) of a given term in an ontology. The IC of a term *t* is defined by the negative log-likelihood in Eq. (10).

10 $$ IC(t)=-\log p(t)  $$

where *p*(*t*) is the probability of encountering an instance of the term *t*. According to this information, Resnik similarity is defined as

11 $$ {sim}_{Resnik}(t_{1},t_{2})=-\log p(t_{m})  $$

where *t*_*m*_ is the most informative common ancestor of *t*_1_ and *t*_2_ in the ontology.

Lin similarity [6] is defined as

12 $$ {sim}_{Lin}(t_{1},t_{2})=\frac{2 * \log p(t_{m})}{\log p(t_{1}) + \log p(t_{2})}  $$

Jang&Conrath similarity [5] is instead defined as

13 $$ {sim}_{J\&C}(t_{1}, t_{2})=2*\log p(t_{m}) - \log p(t_{1}) - \log p(t_{2})  $$

simGIC similarity [33] and simUI similarity [34] compute the functional similarity between proteins. Let *T*_1_ and *T*_2_ be the set of GO terms that annotate proteins *p*_1_ and *p*_2_, respectively. simGIC similarity is defined by the Jaccard index as Eq. (14) while simUI is by the universal index as Eq. (15).

14 $$ {fun}_{GIC}(p_{1},p_{2})=\frac{\sum_{t \in T_{1} \cap T_{2}} IC(t)}{\sum_{t \in T_{1} \cup T_{2}} IC(t)}  $$

15 $$ {fun}_{UI}(p_{1},p_{2})=\frac{\sum_{t \in T_{1} \cap T_{2}} IC(t)}{\max\{\sum_{t \in T_{1}} IC(t), \sum_{t\in T_{2}} IC(t)\}}  $$

The three kinds of combinations for Resnik, Lin, and Jang&Conrath similarities include average (AVG), maximum (MAX), and best-match average (BMA), and they are defined by Eqs. (16), (17), and (18), respectively.

16 $$ {} {fun}_{AVG}(p_{1},p_{2})=\frac{1}{|T_{1}||T_{2}|}\sum_{t_{1} \in T_{1}, t_{2} \in T_{2}} IC(\{t_{1}, t_{2}\})  $$

17 $$ {}{fun}_{MAX}(p_{1},p_{2})=\max\{IC(\{t_{1}, t_{2}\})|t_{1}\in T_{1}, t_{2}\in T_{2}\}  $$

18 $$ {} \begin{aligned} {fun}_{BMA}(p_{1},p_{2})&=\frac{1}{2}\left(\frac{1}{|T_{1}|}\sum_{t_{1}\in T_{1}} IC(\{t_{1},t_{2}\})\right.\\ &\qquad+\left.\frac{1}{|T_{2}|}\sum_{t_{2}\in T_{2}}IC(\{t_{1},t_{2}\})\right) \end{aligned}  $$

### Corpus-based word vector methods

Onto2Vec [25] uses the word2vec model [28] with the skip-gram algorithm to learn from the descriptive axioms of GO terms and proteins. Given a sequence of training words *w*_1_, *w*_2_,..., *w*_*K*_, the skip-gram model aims to maximize the average log-likelihood of Function (19),

19 $$ Loss = \frac{1}{K} \sum_{k=1}^{K}\sum_{-S\leq i \leq S, i\neq 0} \log p(w_{t+i}|w_{t})  $$

where *S* is the size of the training text and *K* is the size of the vocabulary. After getting the word vectors from the word2vec model, Onto2Vec linearly combines the word vectors for proteins according to the words appearing in the descriptive axioms of proteins

20 $$ v(p)=\sum_{w_{i}\in W} v(w_{i})  $$

where *v*(*p*) is the vector of protein *p*, *v*(*w*_*i*_) is the vector of word *w*_*i*_, and *W* represents the set of words in the descriptive axiom of protein *p*.

w2vGO [27] also uses the word2vec model to learn word vectors from the descriptive axioms of GO terms. After that, it uses the word vectors to obtain the vectors of GO terms according to the modified Hausdorff distance [43] as described in Eq. (9), and then use the vectors of GO terms to obtain the vectors of proteins according again to the modified Hausdorff distance.

## Data Availability

The datasets that are used in this paper can be found from their links. Gene Ontology (date of visit: 23 June 2018): http://geneontology.org/docs/download-ontology/ GO annotations (date of visit: 23 June 2018): https://www.uniprot.org/ CESSM dataset (date of visit: 30 October 2018): http://xldb.di.fc.ul.pt/tools/cessm/ PPI dataset (date of visit: 30 October 2018): https://string-db.org/cgi/input.pl Our used data are available at https://github.com/xszhong/GO2Vec.

## Abbreviations

AUC: Area under the curve
BP: Biological process
CC: Cellular component
CESSM: Collaborative evaluation of GO-based semantic similarity measures
DAG: Directed acyclic graph
ECC: Enzyme comparison class
GO: Gene ontology
IC: Information content
MF: Molecular function
PPI: Protein-protein interaction
ROC: Receiver operating characteristic
